# Breath Analysis of COVID-19 Patients in a Tertiary UK Hospital by Optical Spectrometry: The E-Nose CoVal Study

**DOI:** 10.3390/bios13020165

**Published:** 2023-01-20

**Authors:** Steven Laird, Luke Debenham, Danny Chandla, Cathleen Chan, Emma Daulton, Johnathan Taylor, Palashika Bhat, Lisa Berry, Peter Munthali, James A. Covington

**Affiliations:** 1 University of Coventry and Warwickshire Hospital Trust, Clifford Bridge Road, Coventry CV2 2DX, UK; 2 Coventry and Warwickshire Pathology Service, University of Coventry and Warwickshire Hospital Trust, Clifford Bridge Road, Coventry CV2 2DX, UK; 3 Warwick Medical School, University of Warwick, Coventry CV4 7AL, UK; 4 School of Engineering, University of Warwick, Coventry CV4 7AL, UK

**Keywords:** breath analysis, COVID-19, SARS-CoV-2, electronic nose, optical spectrometry

## Abstract

Throughout the SARS-CoV-2 pandemic, diagnostic technology played a crucial role in managing outbreaks on a national and global level. One diagnostic modality that has shown promise is breath analysis, due to its non-invasive nature and ability to give a rapid result. In this study, a portable FTIR (Fourier Transform Infra-Red) spectrometer was used to detect chemical components in the breath from Covid positive symptomatic and asymptomatic patients versus a control cohort of Covid negative patients. Eighty-five patients who had a nasopharyngeal polymerase chain reaction (PCR) test for the detection of SARS-CoV-2 within the last 5 days were recruited to the study (36 symptomatic PCR positive, 23 asymptomatic PCR positive and 26 asymptomatic PCR negative). Data analysis indicated significant difference between the groups, with SARS-CoV-2 present on PCR versus the negative PCR control group producing an area under the curve (AUC) of 0.87. Similar results were obtained comparing symptomatic versus control and asymptomatic versus control. The asymptomatic results were higher than the symptomatic (0.88 vs. 0.80 AUC). When analysing individual chemicals, we found ethanol, methanol and acetaldehyde were the most important, with higher concentrations in the COVID-19 group, with symptomatic patients being higher than asymptomatic patients. This study has shown that breath analysis can provide significant results that distinguish patients with or without COVID-19 disease/carriage.

## 1. Introduction

There has been increased interest in the use of volatile organic compound (VOC) and inorganic gas detection in the diagnostic assessment of infection since the beginning of the SARS-CoV-2 (COVID-19) pandemic [1]. Technologies such as lateral flow tests (LFTs) or polymerase chain reaction (PCRs) detection have been adopted to detect the presence of SARS-CoV-2 RNA from nasal and pharyngeal swabs to manage COVID disease and to prevent onward transmission of the virus. These tests not only vary in cost and availability but also in sensitivity and specificity [2,3]. Most of all, patients can find a nasal, pharyngeal or combined nasopharyngeal sampling uncomfortable, and a poor sample may lead to a false negative result.

VOC/inorganic gas analysis is used extensively in several industrial settings. For example, in the food industry, this technology is used to detect the presence of pesticides and degrading produce [4,5]. In addition, the chemical industry uses this technology to monitor air pollution from the gases released back into the atmosphere [6,7]. However, its use for medical diagnostics and monitoring has become an active area of research [8,9,10]. A number of papers have been published that have studied the potential of breath analysis to detect the presence of SARS-CoV-2 in patients at the point of care or within a clinical setting. These predominantly used commercial platforms and are based on a wide range of technologies. Some can be considered traditional electronic noses and consist of an array of metal-oxide gas sensors combined with smart pattern recognition software. This includes the Aeronose (The Aeonose Company, Netherland) and the SpiroNose (Breathomix, Netherlands), as well as prototype systems [11,12,13]. Other groups used nanomaterials their electronic nose systems [14]. Another regularly used technology is ion mobility spectrometry (IMS), combined with pre-separation based with on GC (gas chromatograph) using a capillary column or MCC (multi-capillary column). These have shown promise for the detection of COVID-19 biomarkers in breath [15,16,17]. There have been further studies using tools such as GCMS (gas chromatography mass spectrometry) and PTR-MS (proton transfer reaction—MS), both of which have identified potential biomarkers for COVID-19 in breath. However, these are more challenging to use as a point of care test due to the size and cost of the instruments, making them more a research tool [18,19].

Though both electronic nose and IMS technologies have been applied to COVID-19 detection in breath, optical techniques, such as FTIR (Fourier Transform Infrared) is also a highly promising approach. These predominantly operate by measuring the absorbance by a gas or a VOC at a specific wavelength and are used extensively for point of care measurements in a wide range of fields [20], including medical [21]. A recent study carried out by Sholomo et al. indicated that FTIR has the potential for COVID-19 diagnosis in breath [22]. Though this result was very positive, the size of the instrument was considerable (non-portable), did not consider systematic and asymptomatic patients, and the FTIR was not used to monitor any bio-marker concentrations. Furthermore, this study was not undertaken in the UK and patients were not tested at admission. The aim of the ENOSE CoVal study was to use a fully portable FTIR instrument and to take it into a major UK hospital to correlate breath gases/VOCs with a clinical presentation, and secondly, to investigate inorganic/VOC differences in patients’ breath between COVID-19 positive (asymptomatic/symptomatic) patients and COVID-19 negative patients.

## 2. Materials and Methods

### 2.1. Patient Group

Patients were recruited from University Hospital Coventry and Warwickshire (UHCW), an 1100 bed tertiary UK hospital in the West Midlands, UK. The COVID-19 positive, symptomatic group were patients that were admitted based on respiratory symptoms and were then found to be COVID-19 positive based on a PCR test. Asymptomatic patients were identified through regular SARS-CoV-2 screening in accordance with the national guidelines at the time. COVID-19 negative patients (control arm) were identified from patients entering hospital for surgery associated with non-infectious conditions (for example, a prostate biopsy). These patients received a SARS-CoV-2 PCR test prior to admission to the hospital. The patients were reviewed for eligibility, consented to enter the study and were given a patient information leaflet. A letter detailing recruitment was then sent to their general practitioner. Demographic data collected included the age, gender, nationality, smoker status, medications they were currently prescribed and SARS-CoV-2 vaccination status of the patient. The most common medications the participants were prescribed were paracetamol, bisoprolol, omeprazole, AdCal D3, a statin, metformin, amlodipine, ramipril and sertraline. These drugs were seen in all the groups. In total, 85 patients were recruited into the study. Table 1 provides the demographic breakdown of the patient groups.

### 2.2. Breath Sampling

The sample was only taken if the patient had not eaten, smoked tobacco, vaped nicotine or drunk anything but water for 3 hours. A demonstration was given to each participant, showing the optimal manner of breathing into the 1.5 litre collection bags.

### 2.3. Breath Analyser

**Figure 1:**
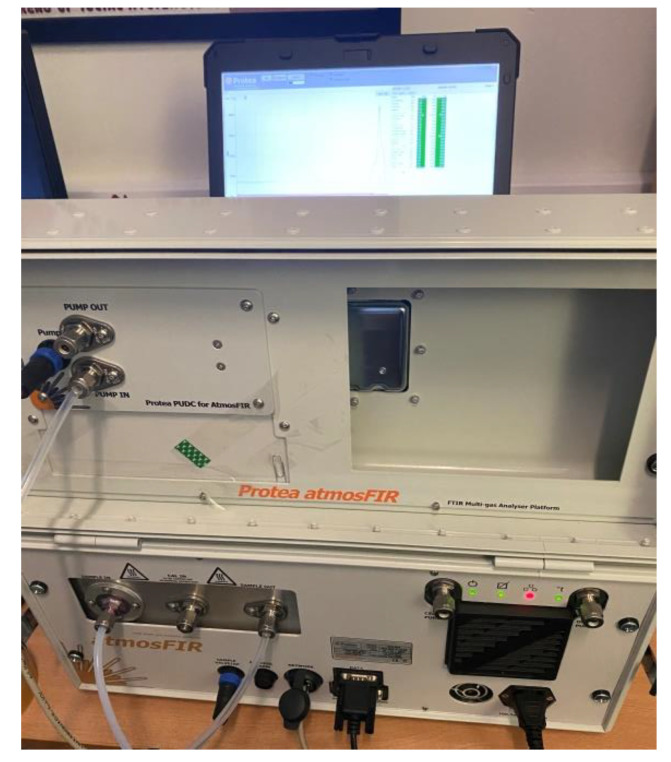
The Protea atmosFIR gas analyser.

In this study, a commercial FTIR (Fourier-transform infrared spectrometer) was used (Figure 1). This was chosen due to its ability to measure gas phase samples in real-time with the ability to detect a large number of inorganic gases and VOCs. Furthermore, it has the potential to provide concentration information on specific VOCs. The unit chosen was a AtmosFTIR platform from Protea (UK). This unit is designed to measure stack emissions and ambient air monitoring and thus is portable, just requiring a power plug to operate it, and was small enough to fit onto a standard medical trolley. The unit is designed to sweep optical frequencies with a spectral range of 485–8500 cm^−1^. It weighs around 21 Kg and, depending on the gas, has a detection limit below 0.2 ppm (parts per million). The unit was left to warm and was calibrated in pure nitrogen before the start of the experiments, in line with the recommendations from the manufacturer. Furthermore, before each batch of experiments, a room air sample was taken to ensure that the machine was functioning correctly and to ensure that the room was not heavily contaminated with other gases/VOCs. Data were collected using a laptop connected to the instrument, and saved locally on the hard disc. The FTIR was calibrated by the manufacturer for the following compounds: acetaldehyde, acetone, ammonia, benzene, carbon monoxide, carbon dioxide, chloroform, ethanol, ethylene oxide, formaldehyde, hydrogen bromide, hydrogen cyanide, hydrogen sulphide, methane, methanol, methyl mercapatan, nitric oxide, nitrogen dioxide, nitrous oxide, ozone, phosphine, sulphur dioxide, and toluene.

The instrument was located in a room close to the ward and samples were transported from the patient to the instrument within a few minutes of collection. Patients consented prior to sampling, and 1.5 L of true tidal volume was captured into a Tedlar sampling bag (Thames Restek, High Wycombe, UK). This volume was used as the analytical cell in the FTIR was 300 mL (excluding valves/pipes etc.) and ensured that the cell could be completely filled with patient breath before the bag was empty. Furthermore, the large volume of breath also minimized changes in pressure sampling through the transfer process. The FTIR was fitted with shutoff valves, both on the inlet and outlet, to seal in a test sample. The bag was attached to the FTIR using tubing, with all valves open. Once attached, the sample was pulled directly into the machine, taking less than 1 min. After this, the valves on the sample inlet and pump outlet were closed and the bag removed. This allowed the breath sample to be held in the sample chamber for the full sampling period of 6 min. The FTIR had a cell temperature setting of 40 °C, and a nominal gas flow of 2.5 L/min. A spectra was generated every minute, comprising of 5 scans (12 s each) averaged together to give the resultant spectra. Once the sample was completed, the valves were opened and at least 8 min was left between samples to ensure there was no sample carryover from the previous patient. In addition, a new Tedlar bag was used for each experiment.

For QA/QC checks, the instrument had to be operating correctly before a sample was measured. This included flow rates, temperature zones and checking that all internal settings were correct. Next, the output of the instrument in room air was checked for any contamination (such as cleaning fluids) before a sample was measured. This was done by checking the chemical outputs of the instrument before analysis. Furthermore, the unit was referenced to pure N_2_ when possible, within the limitations of the instrument being in a restricted area.

### 2.4. Data Analysis

The instrument provides two different outputs. The first provides a list of concentrations (in ppm) for a common set of 16 chemicals. The second is the raw datafiles of the IR spectra, containing 8500 data points per scan. The instrument is free running with a scan rate of 1 Hz. The instrument averages 10 scans before recording this scan to a file. Each created multiple files per scan. The CO_2_ levels were monitored throughout the scan period and the scan with the highest CO_2_ reading was used for further data analysis. Due to the dynamics of the internal chamber, this was likely to align with the highest concentration of gases and VOCs.

The raw data was first checked for sample alignment, and corrections were made if necessary. The data was then thresholded to reduce the dimensionality. This value was chosen to be twice the background level and the same threshold was applied to all the samples. This reduced the number of data points to 3500. These were then treated as independent features and were used in a cross-validation approach. First, the data was divided into 10 groups, with 9 being used for training and the 10th for the test. Within the training group, a rank-sum test was applied to find the 100 features with the lowest *p*-value. These were then used to create two models, sparse linear regression (SLR) and random forest (RF), which were then applied to the test group. This was repeated 10 times until all the data had been a test group. From these results, statical parameters were calculated including AUC (area under the curve), sensitivity, specificity, positive predictive value and negative predictive value. This was undertaken using a custom R script and is similar to the pipeline we have used in other studies [23,24].

In addition, the absolute identified chemicals were also processed. In this case, the value was used as the input feature. Chemicals that would not be found naturally in the body (or in the environment) or were not present in the majority of samples were removed from the list before processing. The excluded chemicals included chloroform, ethylene oxide, formaldehyde, hydrogen bromide, ozone, nitrogen dioxide, nitric oxide and phosphine. This left acetaldehyde, acetone, ammonia, benzene, carbon monoxide, carbon dioxide, ethanol, hydrogen cyanide, hydrogen sulphide, methane, methanol, methyl mercapatan, sulphur dioxide and toluene. The shortlist of chemicals was then used as input features for classification and a similar cross-validation approach, as described above, was used.

## 3. Results

**Figure 2:**
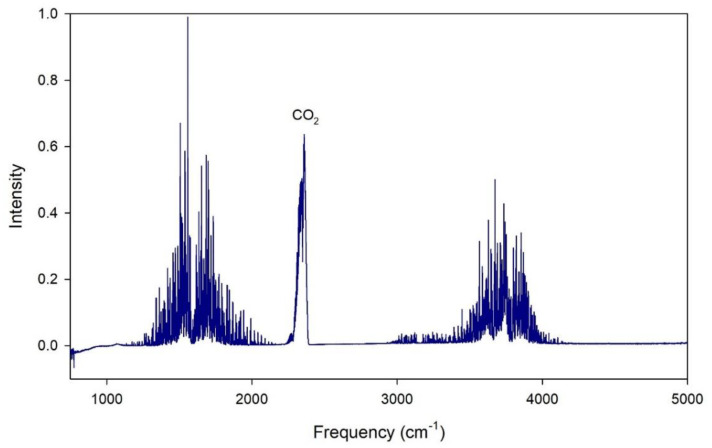
Typical spectral output of a COVID-19 positive patient.

A typical raw output plot of an asymptomatic COVID-19 patient is shown in Figure 2. As can be seen, much of the output is dominated by the water peaks produced by the high humidity in human breath. However, it shows that a high information odour content was collected from each sample. We have directly labelled the CO_2_ peak for reference.

**Figure 3:**
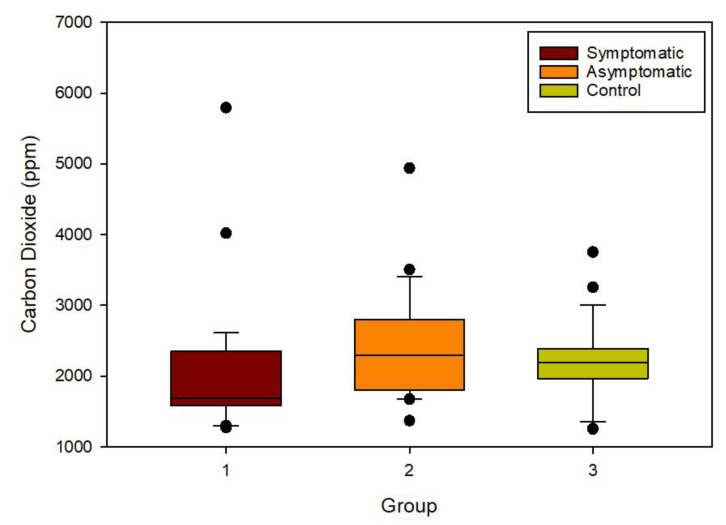
Carbon dioxide levels measured for different patient groups.

Figure 3 shows the CO_2_ levels for all the samples at which the data was used for further data analysis. Though there is variance across all the sample groups, every group is similar. The results indicated that the symptomatic patients had the lowest CO_2_ concentration, which may be linked to the severity of the disease and ability to breathe. However, the chemical information content of these samples remained high.

**Figure 4:**
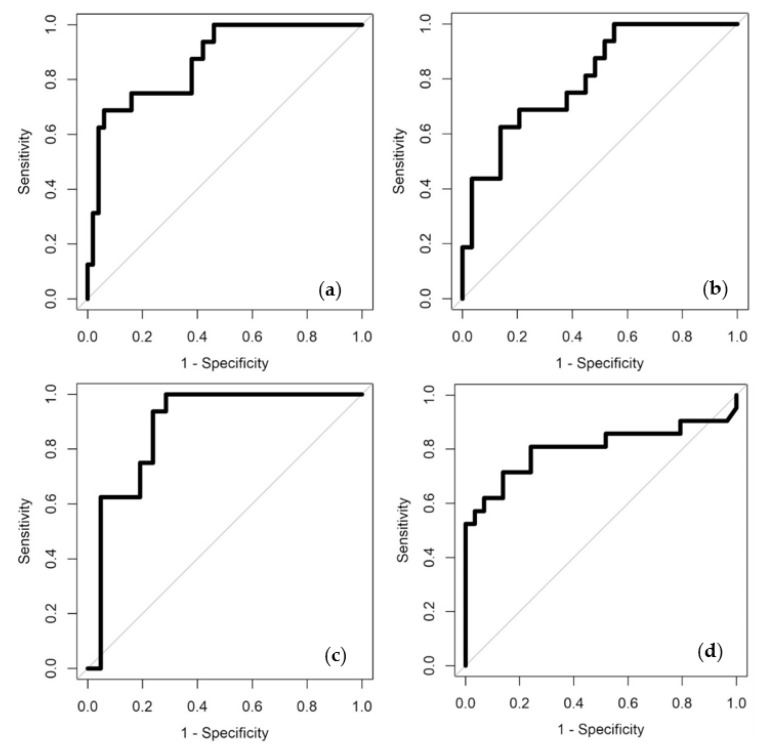
ROCs for patient group combinations (**a**) COVID-19 positive (symptomatic and asymptomatic) vs. COVID-19 negative (control) (**b**) COVID-19 positive (symptomatic) vs. COVID-19 negative (control), (**c**) COVID-19 positive (asymptomatic) vs. COVID-19 negative (control) and (**d**) COVID-19 positive (symptomatic) vs. COVID-19 positive (asymptomatic).

The analysis of the data and statistical results are presented in Table 2, with the subsequent ROCs in Figure 4.

We see that all the results show a statistically significant difference between the groups, with COVID-19 positive (symptomatic and asymptomatic) vs. COVID-19 negative (control) producing an AUC of 0.87. Similar results were obtained comparing COVID-19 positive (symptomatic) vs. COVID-19 negative (control) and COVID-19 positive (asymptomatic) vs. COVID-19 negative (control). However, it was found that the Covid positive (asymptomatic) results were higher than the symptomatic (0.88 vs. 0.80 AUC). Furthermore, we were able to distinguish asymptomatic and symptomatic patients with a high degree of confidence, suggesting that breath VOCs can measure disease severity.

**Figure 5:**
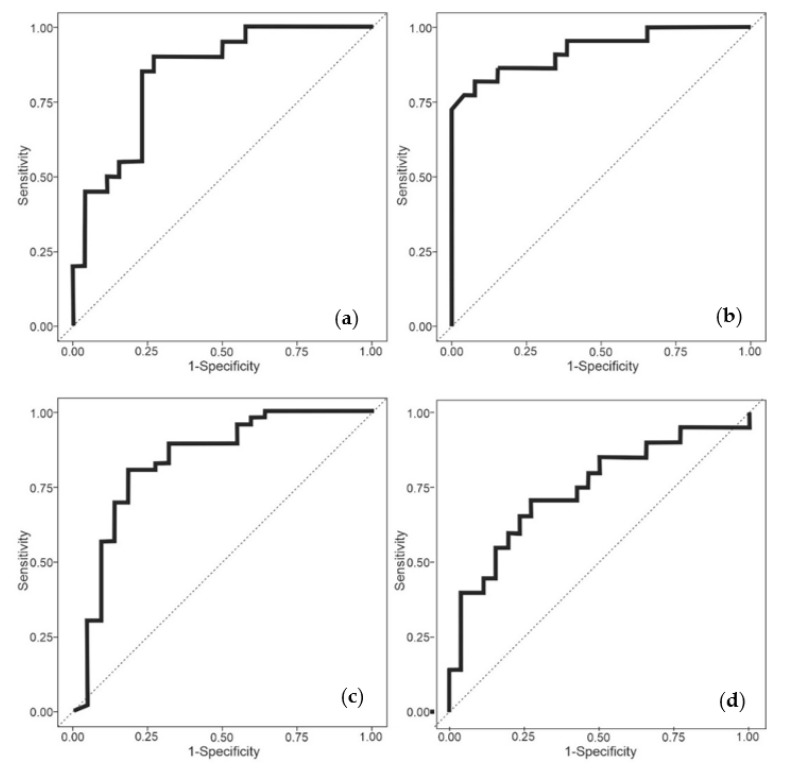
ROCs for patient groups combinations (**a**) COVID-19 positive vs. Control (**b**) Symptomatic vs. Control, (**c**) Asymptomatic vs. Control and (**d**) Symptomatic vs. Asymptomatic.

We also considered the individual chemicals to evaluate whether these gave better separation over taking the data as a whole. In this case, each chemical was used as a feature. The statistical results are shown in Table 3, with the associated ROCs in Figure 5.

Using individual chemicals, we achieved an almost identical result for COVID-19 positive versus controls to that which was achieved when using all the data. However, with this approach, symptomatic patients were identified with a much higher degree of selectivity compared to asymptomatic. This difference in result could well be linked with the way that features were identified in the large feature set.

**Figure 6:**
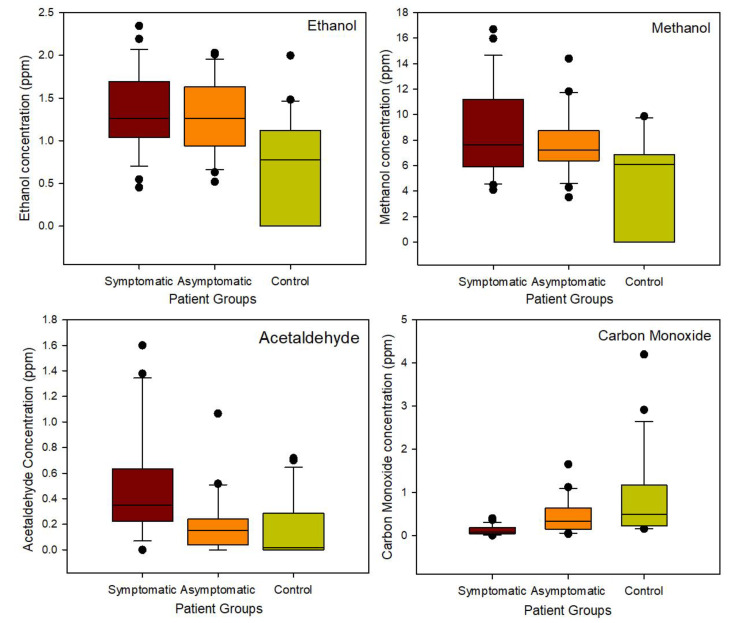
Concentration of four discriminatory chemicals for the patient groups.

From this analysis, we were able to identify the chemical components that separated the data. In this case, the top four chemicals were ethanol, methanol, acetaldehyde and carbon monoxide. Figure 6 provides a box plot of the concentrations of these chemicals for the three patient groups.

As can be seen in Figure 5, in the case of the first three chemicals, the chemical concentrations are highest in the COVID-19 group, with symptomatic patients being higher than asymptomatic patients. The exception is for carbon monoxide, which could potentially be an external contamination factor as control patients enter the hospital and is from the surrounding environment.

## 4. Discussion

The technology used in this study has been shown to significantly differentiate between patients with COVID-19 positive (symptomatic) disease, COVID-19 positive (asymptomatic) carriers and those who do not have the SARS-CoV-2 virus present in the respiratory tract (control). The manner in which the United Kingdom and the global community have managed COVID-19 has evolved over the last two years but the need for non-invasive testing of respiratory viruses is ever present. These breath samples, which are processed on an FTIR, can be simply taken from patients rather than using an uncomfortable nasopharyngeal swab, thus enhancing a patient’s experience. Portable devices are currently in development and being trialled using odour analysis technology, however, this is the first study which offers information on the optical spectrometry profile of the breath in COVID-19 positive (symptomatic and asymptomatic) versus COVID-19 negative patients.

Technologies such as the matrix-assisted laser desorption/ionisation time of flight mass spectrometer is now incorporated in many UK laboratories. This technology in many settings is the primary means of identification of many microbes using a protein signature. Although this technology is markedly different in its mechanism of action, it also requires a substantial database of information to identify the species of the bacteria or fungi it is processing. The same principle will likely be required for this optic scanning technology.

VOCs are present in humans when there is illness and thus physiological stress. Therefore, this is one important marker of disease, but we see this in different hierarchies with the other compounds deemed the most important in differentiating disease and non-disease states. A review from 2013 highlighted a number of different inorganic/VOCs which were prevalent in the patients either infected or colonised with a number of different micro-organisms such as higher concentrations of hydrogen cyanide in the breath of those patients with pseudomonas aureginosa colonisation in their respiratory tract [25].

Methanol, ethanol and acetaldehyde have also been shown to be important markers differentiating the clinical groups within this study, which were also identified in a previous study [16]. Our best results achieved an AUC of 0.93 for symptomatic vs. controls and 0.84 for all COVID-19 subjects versus controls, which is a similar diagnostic performance to other work in breath analysis studies [13,14,16,17,18,19]. These different concentrations of the inorganic/VOCs have also been able to allow us to identify those patients who have symptomatic disease versus asymptomatic disease. This is likely due to the oxidative stresses occurring in the respiratory tract at the time of sampling. The more samples processed and the more profiling documented, the more robust the identification of disease will be.

Previous trials using similar technology have been performed and the results have been positive, but only through collaboration and data compilation will profiling be achieved. Work in the past has focussed on infectious processes such as pneumonia, aiming to identify the difference between a bacterial pneumonia and a viral pneumonitis [26]. These studies have shown promise. Other infection studies have focussed on tuberculosis and enteric pathogens leading to colitis [27,28]. However, breath technology has also been used to identify cancers [29]. This year, we have seen further work of trained medical detection dogs, which have been trained to identify patients positive with COVID-19 [30], but there are limitations to training animals when aiming to provide a reproducible technology on a mass scale.

Therefore, the e-NOSE CoVal study may be a way of diagnosing disease in primary and secondary care. If we focus on the diagnosis of infectious diseases, the benefits of rapid, cost-effective diagnostics will lead to improved infection and prevention control for those transmissible diseases such as COVID-19, reduced antibiotic consumption for viral, not bacterial diseases and a better outcome for the individual patient. If antibiotic stewardship is supported by the eNOSE technology then this not only will negate any side effects experienced by the individual patient who may be prescribed unnecessary antibiotics, but it will also reduce the selective pressures bacteria undergo when antibiotics are used, thus reducing antibiotic resistance in the surviving biome or the pathogenic bacterial population.

The study has limitations. First, as with other eNOSE technologies, the environment, i.e., the gases of the room, may affect the composition of the sample. Ideally, a well-ventilated room should be used, and strong cleaning products should not have been used recently. Secondly, the patient’s own breath composition may be affected by recent food consumption, or strong fluids such as coffee or tobacco use. We have reduced this confounder by not assessing any patient who had eaten or smoked 3 h prior to collecting their sample. Thirdly, the technology offers a range of identified chemical components, but the list is not exhaustive and other gaseous products in the breath may have relevance. Finally, although we believe the number of people enrolled into this study to be appropriate, the sample size still remains low, and the participants will have had other medical problems which may affect their breath components. This has been acknowledged in our statistical analysis.

## 5. Conclusions

In this paper, we report on the use of an FTIR for the analysis of breath samples from COVID-19 positive patients and controls. Here, a commercially available, portable FTIR system was modified to accept breath samples. This study has proven that breath analysis can be used in a tertiary hospital setting to identify SARS-COV-2 infection and colonisation. The technology produces rapid results and the analyser is simple to use. This study further supports electronic nose/breath analysis technology as viable diagnostic test that can be used in a healthcare setting.

## Figures and Tables

**Table 1:** Demographic data for subject groups.

Group	Symptomatic	Asymptomatic	Controls
**Number of samples and percentage**	36 (42.4%)	23 (27.1%)	26 (30.5%)
**Mean Age (years)**	56.7	66.7	53.3
**Gender; Male/Female**	20:16	14:9	20:6
**Vaccinated**	26 (72.4%)	21 (91.3%)	24 (92.3%)
**Nationality**			
British	24 (66.7%)	18 (78.2%)	19 (73.1%)
Caribbean	0	1 (4.35%)	0
Indian	1 (2.7%)	0	3 (11.5%)
Iranian	3 (8.4%)	0	0
Irish	1 (2.7%)	2 (8.7%)	1 (3.85%)
Latvian	0	0	2 (7.7%)
Pakistani	0	1 (4.35%)	0
Portuguese	1 (2.7%)	0	0
Somalian	0	1 (4.35%)	0
Unknown/not declared	6 (16.8%)	0	1 (3.85%)
**Smoking History**			
Current Smoker	2 (5.6%)	4 (17.4%)	11 (42.3%)
Ex-smoker	9 (25%)	5 (21.7%)	4 (15.4%)
Never smoked	25 (69.4%)	14 (60.9%)	11 (42.3%)
**Drug History** Total number of medications taken per group and the average number of medications per patient	160 (4.3 per patient)	72 (3.1 per patient)	50 (1.9 per patient)
Range of the number of medications taken per patient	0–11	0–11	0–7

**Table 2:** Statistical results comparing different patient groups.

Comparisons	Classifiers	AUC	Sensitivity	Specificity	PPV	NPV
COVID-19 positive (symptomatic and asymptomatic) vs. COVID-19 negative (control)	SLR	0.76 (0.61–0.92)	0.69 (0.41–0.89)	0.88 (0.76–0.95)	0.65	0.90
	RF	0.87 (0.77–0.97)	0.69 (0.41–0.89)	0.94 (0.83–0.99)	0.79	0.90
COVID-19 positive (symptomatic) vs. COVID-19 negative (control)	SLR	0.80 (0.67–0.93)	0.63 (0.35–0.85)	0.86 (0.68–0.96)	0.71	0.81
	RF	0.77 (0.62–0.92)	0.69 (0.41–0.89)	0.86 (0.68–0.96)	0.73	0.83
COVID-19 positive (asymptomatic) vs. COVID-19 negative (control)	SLR	0.83 (0.69–0.97)	0.88 (0.62–0.98)	0.76 (0.53–0.92)	0.74	0.89
	RF	0.88 (0.77–1)	0.88 (0.62–0.98)	0.76 (0.53–0.92)	0.74	0.89
COVID-19 positive (symptomatic) vs. COVID-19 positive (asymptomatic)	SLR	0.78 (0.65–0.92)	0.71 (0.48–0.89)	0.79 (0.60–0.92)	0.71	0.79
	RF	0.80 (0.66–0.95)	0.71 (0.48–0.89)	0.86 (0.68–0.96)	0.79	0.81

**Table 3:** Statistical results comparing different patient groups using individual chemicals.

Comparisons	Classifiers	AUC	Sensitivity	Specificity	PPV	NPV
COVID-19 positive (symptomatic and asymptomatic) vs. COVID-19 negative (control)	SLR	0.88 (0.80–0.95)	1.00 (1–1)	0.50 (0.32–0.68)	0.80	1.00
	RF	0.84 (0.74–0.93)	0.89 (0.81–0.96)	0.50 (0.33–0.69)	0.79	0.69
COVID-19 positive (symptomatic) vs. COVID-19 negative (control)	SLR	0.91 (0.83–0.98)	0.68 (0.50–0.83)	0.96 (0.89–1)	0.94	0.78
	RF	0.93 (0.85–0.98)	0.82 (0.67–0.95)	0.85 (0.72–0.96)	0.81	0.85
COVID-19 positive (asymptomatic) vs. COVID-19 negative (control)	SLR	0.76 (0.62–0.88)	0.75 (0.58–0.90)	0.64 (0.46–0.80)	0.65	0.74
	RF	0.74 (0.61–0.86)	0.65 (0.47–0.82)	0.73 (0.58–0.87)	0.65	0.73
COVID-19 positive (symptomatic) vs. COVID-19 positive (asymptomatic)	SLR	0.84 (0.74–0.93)	0.70 (0.52–0.87)	0.77 (0.62–0.90)	0.77	0.84
	RF	0.74 (0.61–0.86)	0.65 (0.47–0.82)	0.73 (0.58–0.87)	0.65	0.73

## Data Availability

The data presented in this study are available on request from the corresponding author. The data are not publicly available due to the limitations of data use defined in the consenting process.
